# PiezoMEMS Fabrication on Flexible Stainless-Steel Substrates

**DOI:** 10.3390/s26072246

**Published:** 2026-04-05

**Authors:** Kae Nakamura, Chi-Luen Huang, Ali Habib Akhyari, Andrea P. Argüelles, Thomas N. Jackson, Susan Trolier-McKinstry

**Affiliations:** 1 Department of Materials Science and Engineering, The Pennsylvania State University, University Park, PA 16802, USA; 2 Materials Research Institute, The Pennsylvania State University, University Park, PA 16802, USA; 3 Department of Engineering Science and Mechanics, The Pennsylvania State University, University Park, PA 16802, USA; 4 School of Electrical Engineering and Computer Science, The Pennsylvania State University, University Park, PA 16802, USA

**Keywords:** flexible MEMS, PMUT, PZT, metal foil

## Abstract

A bottom-up fabrication approach for flexible piezoelectric micromachined ultrasound transducer (PMUT) arrays on stainless-steel substrates was developed. Devices were fabricated using chemical solution deposition of a 700 nm-thick layer of Pb_0.99_□_0.01_(Zr_0.52_Ti_0.48_)Nb_0.02_O_3_, where □ denotes a vacancy on the Pb site, on 50 μm-thick LaNiO_3_/HfO_2_/stainless-steel foils. Lithography for definition of the electrode and piezoelectric layers was completed on the front of the wafer. Ni electroplating on the back side of the foil was used to create locally stiff areas to define the deflection area. PMUT devices were successfully fabricated using this method. The permittivity and loss tangent of the fabricated device at 1 kHz were 283 ± 9 and <1.5%, respectively. The remanent polarization was measured to be 38 ± 0.3 μC/cm^2^.

## 1. Introduction

Piezoelectric Microelectromechanical Systems (piezoMEMS) are MEMS devices that incorporate piezoelectric materials, offering significant advantages such as large displacement at low driving voltages and facile array integration within a single process flow. Among these, Piezoelectric Micromachined Ultrasound Transducers (PMUTs) represent a well-established class of piezoMEMS devices. PMUTs are typically fabricated on Silicon on Insulator (SOI) wafers. In these structures, the diaphragm membrane is formed by Deep Reactive Ion Etching (DRIE) from the backside of the wafer [1]. As a result, the active region of the PMUT contains a significantly thinner silicon layer than the rest of the wafer, enabling localized bending and control of the device resonant frequency. A comparable technology is Capacitive Micromachined Ultrasonic Transducers (CMUT), which employ a capacitor that consists of a thin conducting membrane suspended over a cavity and utilizes electrostatic force for deflection of the membrane. Compared to PMUTs, CMUTs generally exhibit higher bandwidths and electromechanical coupling coefficients, but require significantly higher driving voltages [2]. This makes PMUTs advantageous for some biomedical applications; the low driving voltage is useful for drive/receive circuitry, and can be critical for wearables. Consequently, PMUTs have been actively explored for applications such as biomonitoring and neuromodulation [3,4,5,6,7,8]. Their capacity to produce large displacements with limited voltage makes them attractive for such use cases.

PMUTs fabricated on SOI wafers rely on a rigid silicon layer as an integral part of the final device structure. However, fabricating PMUTs on flexible substrates presents opportunities for applications that demand mechanical flexibility, high fracture toughness, and enhanced durability. While polymer-based substrates have been employed for such purposes [9,10,11], the use of flexible metal foil substrates is also promising for biomedical applications and nondestructive testing on curved surfaces [12,13,14].

Several studies have demonstrated the feasibility of piezoelectric thin films on nickel or stainless-steel substrates for flexible piezoMEMS devices. For example, Kanno et al. fabricated self-powered PZT-based card-type piezoelectric energy harvesters by sputtering PZT thin films onto ferritic stainless-steel foils with bottom electrodes [15,16,17]. These devices successfully generated up to 20 μW output power when an impulsive force was applied to the energy harvester with a hammer. The resonance frequency of the harvester was approximately 24.5 Hz at an acceleration of 0.3 m/s^2^. Other studies have employed aerosol deposition [18,19,20,21] or sol–gel deposition [22] of PZT onto stainless steel for energy-harvesting applications. A study utilizing bimorph PZT films on Ni substrate was successful in generating a maximum power of 149 μW resonating at 6 Hz via a piezoelectric compliant mechanism [23]. Altogether, these results demonstrate the strong potential of metal foil substrates for energy-harvesting MEMS devices. It is notable, though, that these energy harvesters typically entail little microfabrication beyond electrode patterning and attachment of proof masses.

In addition to energy harvesting, PZT thin films on stainless steel have also been investigated for biomedical sensing. For instance, one study fabricated a device capable of detecting arterial movements by conformally attaching PZT-on-stainless-steel structures to vascular surfaces [24]. Similarly, stainless-steel substrates have been used to realize ultrasonic transducer arrays. One group reported a PZT-based ultrasonic transducer array on stainless steel that achieved successful pulse-echo operation in water over an operating frequency range of 2.4 to 13.8 MHz. The design was notably simple, requiring no photolithographic steps, and employed sol–gel derived PZT films with a painted silver layer serving as the top electrode [25]. In another example, stainless steel served as a substrate for insole-embedded gait sensors, where sol–gel-derived PZT films were directly deposited onto the metal surface. This device exhibited a linear response with a sensitivity of −9.76 mV/N between 0 and 12 N and successfully demonstrated the wireless transmission of digitized pressure-sensing data [26].

Functional PMUT arrays directly fabricated on other metal foils have also been demonstrated. Feng et al. reported PMUTs on 5 μm-thick titanium foils using a hydrothermal method to deposit PZT films on both sides of the foil, and stereolithographic 3D printing to construct supporting structures with resins [27]. Building on this work, Wang et al. developed a bottom-up PMUT fabrication process on titanium foil. In that case, PZT was grown hydrothermally and a cavity was subsequently defined using a SU-8 negative photoresist. In this design, the titanium foil served simultaneously as the substrate and bottom electrode [28]. However, the fabricated devices remained relatively large, with a diameter of 1.80 mm, and exhibited resonance frequencies of 19–20 kHz. Further miniaturization is required to achieve the higher resonance frequencies needed for medical and industrial sensing applications.

These studies demonstrate the potential of metal-foil substrates as platforms for flexible piezoMEMS devices. Compared to polymer substrates, metal foils offer superior thermal stability and mechanical durability, while still providing the flexibility needed for wearable, biomedical, and structural applications. Research to date demonstrates that PZT thin films on metal foils can not only enable efficient energy harvesting, but also support conformal biomedical sensors and functional ultrasonic transducer arrays. These advances collectively suggest that flexible metal foils represent a promising route toward robust, high-performance piezoMEMS that can operate in environments and geometries beyond the reach of conventional silicon-based devices.

Lead zirconate titanate (PZT) films on metal foils also provide an alternative to polyvinylidene fluoride (PVDF) when higher piezoelectric coefficients are needed. In particular, niobium-doped PZT with the composition [Pb_0.99_□_0.01_(Zr_0.52_Ti_0.48_)Nb_0.02_O_3_, where □ denotes a vacancy on Pb site] (PZT) at the morphotropic phase boundary has high piezoelectric coefficients, dielectric constants, and remanent polarization values [29]. Direct deposition of PZT onto metal foil substrates enhances the transverse piezoelectric coefficients (*e*_31,*f*_) relative to films on Si by approximately |2.7| C/m^2^, which is critical for achieving high-output PMUT performance [30]. This enhancement is primarily attributed to the thermal expansion coefficient mismatch between the PZT film and the metal substrate. As the film cools from the crystallization temperature, residual compressive stress develops in the PZT layer, promoting the formation of a higher volume fraction of c-domains [31,32]. This domain configuration results in increased remanent polarization, thereby improving the electromechanical performance of the device [33,34]. Therefore, the direct fabrication of PZT on metal foils offers advantages for PMUT applications that require both mechanical flexibility and high piezoelectric output. In this work, stainless steel was selected as the substrate material, owing to its lower susceptibility to plastic deformation during fabrication relative to foils such as Ni [35,36]. Furthermore, austenitic stainless steel is suitable for biomedical applications, as it exhibits excellent corrosion resistance and is not prone to rusting when exposed to moisture or biological fluids [37].

In this study, a bottom-up fabrication process for PMUTs directly on stainless-steel substrates was developed. The piezoelectric layer, consisting of lead zirconate titanate (PZT) at the morphotropic phase boundary (MPB), was deposited onto a 200 nm-thick LaNiO_3_ bottom electrode deposited on stainless steel passivated with 50 nm HfO_2_. To define the actuation region, a rigid Ni support structure was constructed on the backside of the substrate via nickel electroplating. The novelty of this work lies in the miniaturization of the device architecture and the expansion of microfabrication possibilities for PMUTs directly on stainless-steel substrates through the use of photolithographic patterning and improved confinement structures for PMUT arrays.

This report first discusses the selection of Ni as the support structure, based on finite element model simulation results. Then, the step-by-step fabrication process of the PMUT on stainless steel is described. Lastly, PMUT device characterization results are shared and discussed.

## 2. Selection of Support Structure Material

**Figure 1:**
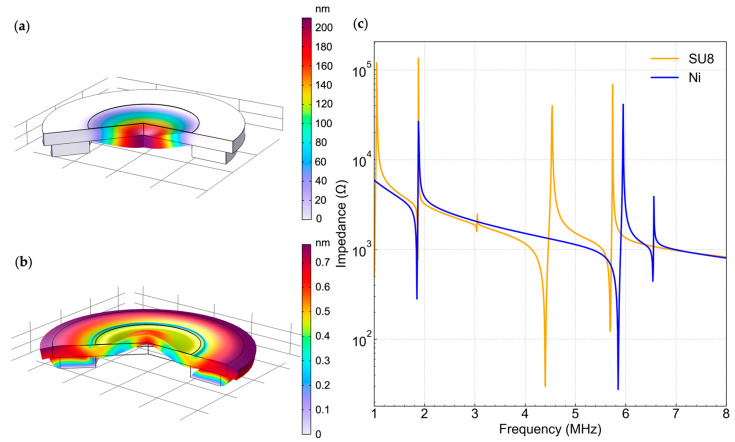
FEM simulated displacement profiles at 1.85 MHz and 1.87 MHz for PMUTs with (**a**) Ni and (**b**) SU-8 support structures, respectively. (**c**) FEM Impedance spectra obtained from a frequency sweep.

The process employs Ni as the rigid structure to confine the deformation area. Ni was selected due to its high stiffness, which provides improved confinement of the vibrating region relative to SU-8, as shown in the Finite Element Model (FEM) simulation results in Figure 1. In the model, a 2D axisymmetric model was utilized for the stainless-steel substrate, the 700 nm PZT layer, the 500 nm Au contact pad electrode, and the Ni support structure. The mechanical constraint was fixed at the bottom of the Ni support structure. A free triangular mesh was utilized, and the mesh density was optimized through convergence analysis, which is detailed in the Supplementary Materials section. Figure 1a and Figure 1b show the displacement profiles of two Finite Element Modeling (FEM) models at 1.85 MHz and 1.87 MHz, respectively, where the impedance sweep results (Figure 1c) exhibit peaks corresponding to the first harmonic resonance.

It is also important to note from the displacement profiles in Figure 1b that SU-8 does not effectively confine the displacement to the diaphragm. Instead, much of the displacement occurs beyond the edges of the polymer layer, suggesting that the polymer will be significantly less effective in reducing cross-talk between elements, as will be required for focusing and beam steering of arrays.

## 3. PMUT Fabrication on Stainless-Steel

**Figure 2:**
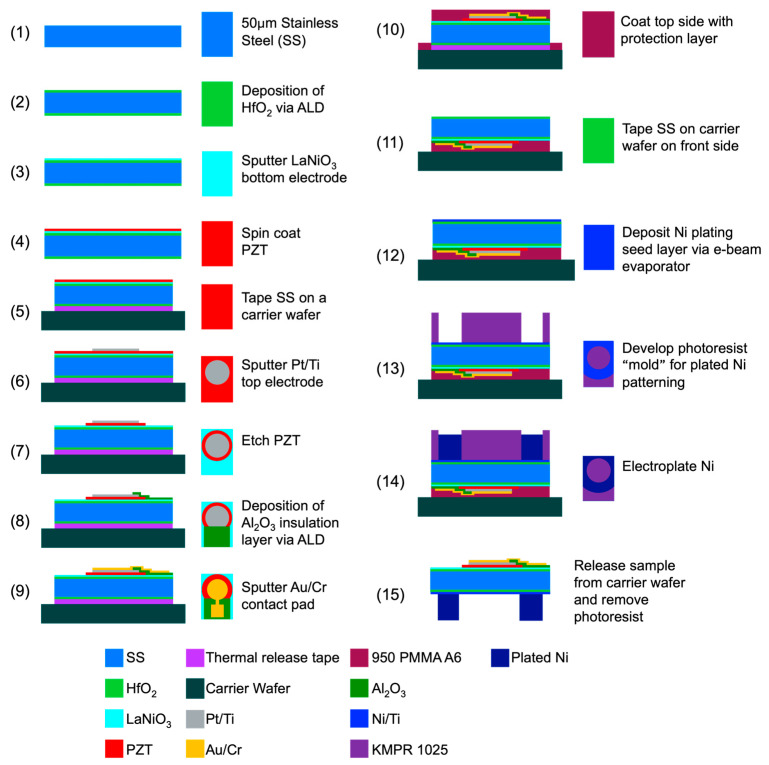
Full fabrication process flow for a PMUT on stainless steel. PMMA = polymethyl methacrylate; KMPR = KMPR series photoresist.

The complete PMUT fabrication process is illustrated in Figure 2, and the thickness of each layer in the device is shown in Table 1. The base substrate was a 5 cm by 5 cm square, 50 μm-thick 304 stainless steel from TDC Corporation. A ~50 nm-thick HfO_2_ passivation layer was grown on both sides of the substrate by atomic layer deposition. Then, 200 nm-thick LaNiO_3_ (LNO) (100)-orienting bottom electrodes were deposited via RF magnetron sputtering from 3″ diameter ceramic targets (Kojundo, Saitama, Japan) using the conditions shown in Table 2, as described previously [38]. LNO was selected as the bottom electrode because it has previously been reported to act as a seed layer for PZT and promote the growth of highly (001)-oriented PZT films on both Si substrates and Ni foils [30]. To fabricate PZT via chemical solution deposition (CSD), a precursor solution was prepared through the inverted mixing order (IMO) preparation method, as described previously [39]. To spin-coat PZT films, a porous ceramic chuck (Cost Effective Equipment, Saint James, MO, USA) was used to mitigate deformation of the flexible stainless-steel substrate. The precursor solution was dispensed onto the substrate and spun at 3000 rpm for 45 s. The film was then pyrolyzed at 100 °C for 1 min and 300 °C for 4 min, followed by crystallization in a rapid thermal annealing system for 1 min at 650 °C. This process was repeated a total of nine times, resulting in a PZT layer thickness of 0.7 μm.

**Figure 3:**
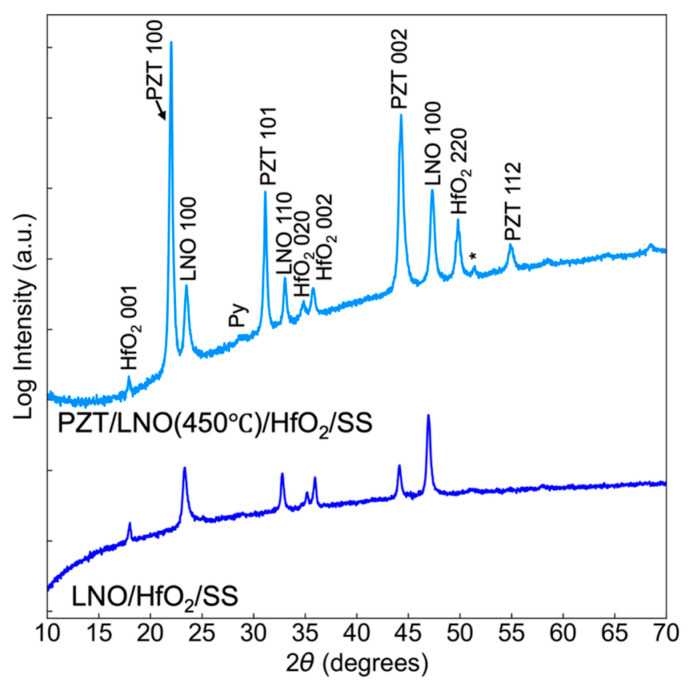
2*θ* XRD scan of before and after deposition of PZT on stainless steel. Substrate peaks are marked with a *. Py = pyrochlore.

Figure 3 shows X-ray diffraction (XRD) data collected using a Malvern Panalytical Empyrean (Malvern Panalytical, Almelo, The Netherlands) with an area detector (PIXcel3D) to examine the crystal structure of the films. The generator voltage during scan was set to 45 kV and the tube current was 40 mA. The scan range in the Bragg–Brentano geometry was 10° to 70° with a step size of 0.0263° with an effective count time of 96.39 s. It was found that the PZT film orientation mimicked that of the underlying LNO as expected. The films were partially {100}-textured, but with other orientations also apparent.

To deposit the top electrode, a MEGAPOSIT^TM^ SPR^TM^ 3012 positive photoresist (DuPont Electronic Materials, Wilmington, DE, USA) was spin-coated at 4000 rpm for 45 s and soft-baked at 95 °C for 1 min. A contact aligner (MA/BA Gen 4, SUSS MicroTec, Garching, Germany) was utilized for all exposures. The top electrode pattern was exposed at 65 mJ/cm^2^. Development was performed using MICROPOSIT^TM^ MF^TM^ CD-26 developer (Kayaku Advanced Materials, Westborough, MA, USA) for 30–60 s. The top electrode was formed by sputtering a 2 nm Ti adhesion layer followed by 100 nm of Pt without breaking vacuum. The photoresist was removed by immersing the sample in PRS 3000 (J.T. Baker, Radnor, PA, USA) at 82 °C. This lift-off method was also used to remove the photoresist after each deposition step during the top-side processing.

To pattern the PZT layer, a 12 μm-thick AZ4620 positive photoresist (Merck, Darmstadt, Germany) was spin-coated at 1500 rpm for 30 s and soft-baked in two steps: 90 °C for 2 min and 105 °C for 5 min. Prior to exposure, the sample was rehydrated for at least 1 h. The resist was then exposed to eight separate 100 mJ/cm^2^ doses, with 30 s intervals between each exposure. Development was carried out in 1:4 diluted AZ400K developer (Merck) for 3 min. PZT was etched using a NE-550 ICP etch system (ULVAC, Kanagawa, Japan), with the parameters listed in Table 3 [6].

The etching was performed in 20 s etch cycles with 1 min cooling intervals, requiring a total of 10 min and 40 s to etch 0.7 μm of PZT.

A 100 nm-thick Al_2_O_3_ layer was deposited via atomic layer deposition (ALD) using an ALD-150LE^TM^ system (Kurt J. Lesker, Jefferson Hills, PA, USA). Following deposition, a MICROPOSIT^TM^ S1827 positive photoresist (DuPont Electronic Materials) was spin-coated at 4000 rpm for 45 s, soft-baked at 115 °C for 2 min, and exposed at 150 mJ/cm^2^. The resist was developed in CD-26 developer for 80 s and baked at 110 °C for 5 min. The Al_2_O_3_ layer was wet-etched by immersing the sample in CD-26 developer; 55 min were required to remove the full 100 nm layer.

To form the Au contact pads, nLOF 2020 negative photoresist (Merck) was spin-coated at 3000 rpm for 45 s, soft-baked at 110 °C for 90 s, and exposed at 100 mJ/cm^2^. A post-exposure bake was also conducted at 110 °C for 90 s. The resist was developed in CD-26 for 90–120 s. Subsequently, contact pad was deposited by sputtering 4 nm Cr adhesion layer followed by 500 nm of Au without breaking vacuum.

After the top side processing, as a protective layer for the top-side, a 950 PMMA A6 photoresist (Kayaku Advanced Materials) was spin-coated at 3000 rpm for 45 s and baked at 100 °C for 5 min. The substrate was then released from the carrier wafer and taped face-down onto a transparent glass substrate to begin back-side processing.

To initialize the back-side processing, a seed layer was deposited utilizing an electron-beam evaporator (Temescal FC2000, FerroTec (Tokyo, Japan)), consisting of 5 nm of Ti adhesion layer followed by 100 nm of Ni. The mold for Ni plating was formed using a KMPR 1025 negative photoresist (Kayaku Advanced Materials), which was spin-coated at 1500 rpm for 30 s, soft-baked at 50 °C for 11 h, exposed at 1850 mJ/cm^2^, and post-exposure baked at 50 °C for 2 h. Development was conducted in CD-26 for 2 min, followed by rinsing with running deionized water for 2~3 min to remove any remaining residues. Ni was initially plated at a current density of 0.5 A/dm^2^ for the first hour, then gradually increased to 2 A/dm^2^. The total time for electroplating was 260 min, yielding a total thickness of 50~70 μm [40].

Following successful electroplating, the sample was removed from the carrier wafer by heating the substrate and releasing the thermal release tape. Photoresists were removed from the device by immersing in PRS3000 solvent at 45 °C until all photoresists were removed.

## 4. PMUT Element Characterization

**Figure 4:**
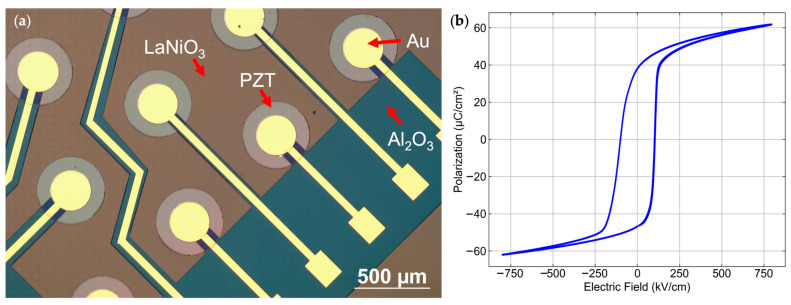
(**a**) Optical microscopic image of a circular PMUT array with 60% top electrode coverage of the released area after Au contact pad deposition. (**b**) Polarization–hysteresis loop for a 0.7 μm-thick PZT film at an electric field of 800 kV/cm.

An image of a circular PMUT array after Au contact pad deposition is shown in Figure 4a. The capacitance and loss of the capacitor structure were measured by sweeping the frequency from 100 Hz to 1 MHz with a 30 mV AC signal using an LCR Meter (4284A Precision, Hewlett Packard (Palo Alto, CA, USA)). The polarization-electric field (P-E) characteristics were subsequently measured using a ferroelectric tester (Multiferroic II, Radiant Technologies, Inc. (Albuquerque, NM, USA)). The permittivity ε_r_ and loss tangent at 1 kHz were 283 ± 9 and <1.5%, respectively. For the P-E loop measurement, the drive signal was set to double bipolar mode, with the maximum field set at 800 kV/cm, and the frequency set at 100 Hz. The resulting hysteresis loop is shown in Figure 4b. The remanent polarization P_r_ was measured to be 38 ± 0.3 μC/cm^2^, indicating good ferroelectric properties of the PZT film.

To determine the piezoelectric coefficient of the structure, the *e*_31,f_ coefficient was measured using the wafer flexure method [41]. Prior to measurement, the device was poled at 150 °C by applying an 18 V DC bias for 15 min, then a 25 V DC bias for 15 min at room temperature. The poling field was selected to be approximately 2–3 times the coercive field, which was measured to be ~100 kV/cm, as shown in Figure 4a. The measured *e*_31,f_ after poling was −4.3 ± 0.3 C/m^2^.

Table 4 summarizes the electrical and electromechanical properties of this work in comparison with prior reports. The reported piezoelectric constants in this work are lower than previously reported values [30,42], due in part to the incomplete texturing of the films. As reported elsewhere, the piezoelectric coefficient *e*_31,f_ values are expected to rise with enhanced film orientation [43,44].

**Figure 5:**
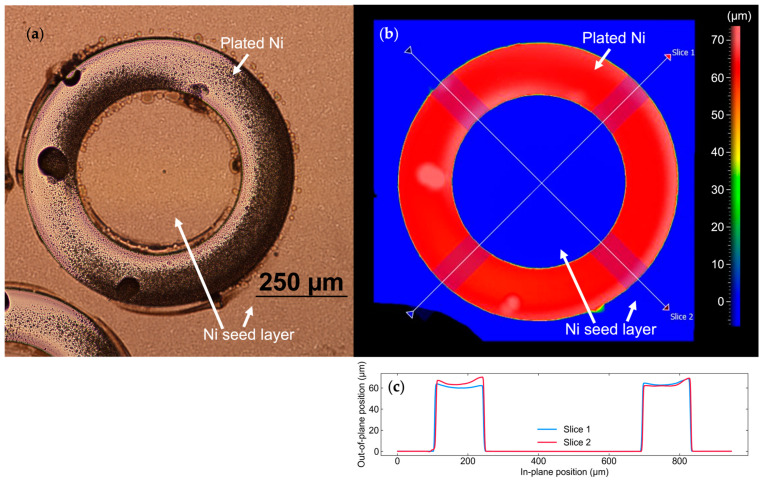
(**a**) Optical microscopic image and (**b**) optical profilometry result of the backside of a circular PMUT element after Ni electroplating and removal of the photoresist “mold”. (**c**) The cross-sectional topography along slices 1 and 2, as indicated in (**b**).

The topography of the backside electroplated Ni was characterized using an optical profilometer (Nexview^TM^ NX2 3D, Zygo (Middlefield, CT, USA)), as shown in Figure 5b,c. The thickness of the electroplated Ni was confirmed to be 63 µm.

**Figure 6:**
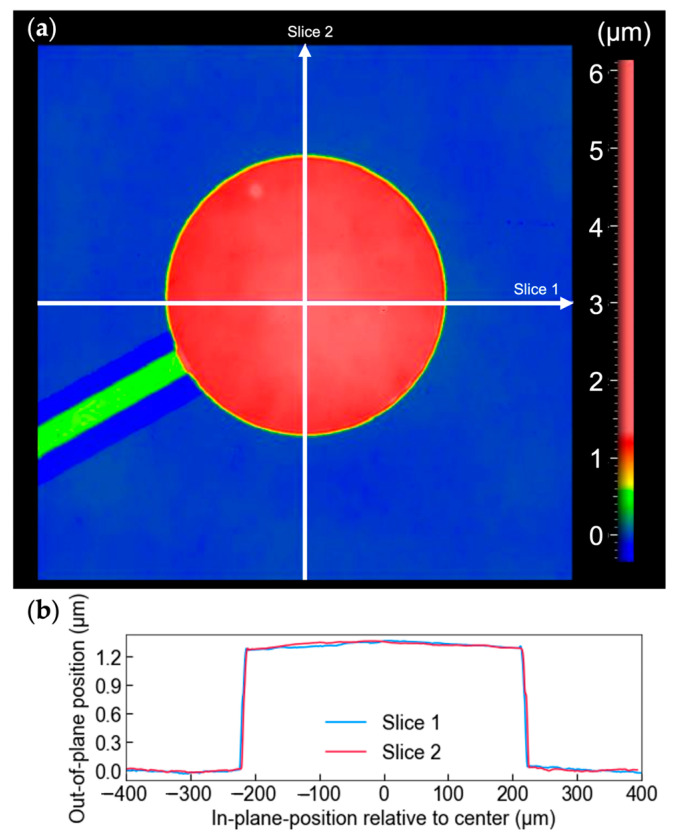
(**a**) Optical profilometry result of the front side of a circular PMUT element. (**b**) The cross-sectional topography along slices 1 and 2, as indicated in (**a**).

To observe the bending mode of the PMUT diaphragm, an optical profilometer was used to scan a single circular PMUT on the front side with a 220 µm radius and a 150 µm-wide Ni support structure. The scan was performed before and after applying a 3 V DC bias between the bottom and top electrode. Prior to measurement, the device was poled at room temperature by applying a 14 V DC bias for 15 min. Figure 6 shows the optical profilometry result at 0 V DC bias of a PMUT element with 100% top electrode coverage of the released area. The observed curvature at the PMUT surface is attributed to residual stress arising from the mismatch in thermal expansion coefficients between the stainless-steel substrate and the deposited PZT layer. Measurement after application of the electric field indicated a maximum additional out-of-plane displacement of 27 nm near the center of the diaphragm.

## 5. Conclusions

Motivated by the need for biomedical applications and nondestructive testing on curved surfaces, a bottom-up fabrication approach was developed for flexible piezoelectric micromachined ultrasound transducer (PMUT) arrays on stainless-steel substrates. A 700 nm-thick chemically deposited PZT film was used as the piezoelectric layer and was deposited directly onto 50 µm-thick LaNiO_3_/HfO_2_/stainless-steel foils. Photolithography was performed on the front side of the substrate to define the electrode and piezoelectric layers. On the backside, Ni electroplating was used to define locally stiffened regions that confine the deflection area. PMUT devices were successfully fabricated using the proposed process.

## Figures and Tables

**Table 1:** Thickness of each layer in the device.

Material	Thickness (µm)
Stainless-Steel	50
HfO_2_ (passivation layer)	0.05
LaNiO_3_ (bottom electrode)	0.2
PZT (piezoelectric layer)	0.7
Pt/Ti (top electrode)	0.1/0.002
Al_2_O_3_ (insulation layer)	0.1
Au/Cr (contact pad)	0.5/0.004
Ni/Ti (plating seed layer)	0.1/0.005
Ni (electroplated structure)	50~70

**Table 2:** LNO sputtering parameters.

Parameters	Range
Power (W)	90
Pressure (mTorr)	7
Target-Substrate distance (inch)	4
Ar/O_2_ flow ratio	3:1
Deposition time (s)	23,600
Substrate Temperature (°C)	450

**Table 3:** Dry etching parameters for PZT using ULVAC NE-550 ICP etch tool.

Parameters	Range
Power (W)	700
Pressure (Torr)	13.0
Ar flow (sccm)	20.0
CF_4_ flow (sccm)	28.0
Cl_2_ flow (sccm)	7.0
Substrate Temperature (°C)	0

**Table 4:** Comparison of PZT thin film properties.

Source	Zr/Ti Ratio	P_r_ (μC/cm^2^)	ε_r_ at 1 kHz	|*e*_31,f_| (C/m^2^)	Film Orientation	Substrate	Film Thickness (µm)
Current work	52/48	38 ± 9	283 ± 9	4.3 ± 0.3	Partially {100} textured	LaNiO_3_/HfO_2_ /SS * 304	0.7
Wilke et al. [41]	52/48	22	1100	6.4	Randomly oriented	Pt/TiO_x_/Si	1.3
Coleman et al. [30]	52/48	32	600 ± 80	9.7 ± 0.45	Highly {100} textured (LF ** = 98%)	LaNiO_3_/Ni	0.6
Minemura et al. [42]	40/60	52	230	-	Highly {100} textured	ns-CN ***/Pt /SS 316L	0.3
Minemura et al. [43]	40/60	60	<300	-	Preferentially {001} oriented	ns-CN/Inconel 625	0.3
Kweon et al. [44]	52/48	14	1180	7.6	Preferentially {001} oriented	SrRuO_3_/Pt/ZrO_2_/Si	1.1–1.4

SS * = Stainless-steel, LF ** = Lotgering Factor, and ns-CN *** = nanosheet Ca_2_Nb_3_O_10._

## Data Availability

The data that support the findings of this study are available from the corresponding author upon reasonable request.

## Supplementary Materials

The following supporting information can be downloaded at: https://www.mdpi.com/article/10.3390/s26072246/s1. Figure S1: Maximum displacement plotted as a function of frequency sweep for models with a different number of elements, for a 220 μm-radius PMUT element with 100% top electrode coverage element and 150 μm Ni support structure width. Table S1: Basic mechanical properties of the materials in the PMUT components. Table S2: Basic mechanical properties of the materials in the PMUT components. Table S3: Basic mechanical properties of the materials in the SU8-support-structure PMUT simulation.

## Abbreviations

The following abbreviations are used in this manuscript:

PMUT	Piezoelectric micromachined ultrasound transducer
piezoMEMS	Piezoelectric microelectromechanical systems
PZT	Pb(Zr,Ti)O_3_
SOI	Silicon on insulator
DRIE	Deep reactive ion etching
CMUT	Capacitive micromachined ultrasonic transducers
PVDF	Polyvinylidene fluoride
FEM	Finite element model(s)
MPB	Morphotropic phase boundary
LNO	LaNiO_3_
CSD	Chemical solution deposition
IMO	Inverted mixing order
