# Supplementary material for: PiezoMEMS Fabrication on Flexible Stainless-Steel Substrates
Source: Sensors (Basel). 2026 Apr 5;26(7):2246. doi: 10.3390/s26072246 (PMC13075221; doi:10.3390/s26072246)
Supplement: Supplementary file 1 [file sensors-26-02246-s001.zip › sensors-4169376-supplementary.pdf]

PiezoMEMS Fabrication on Flexible Stainless-Steel Substrate - Supplementary Materials

1. Finite Element Model Convergence Analysis

A convergence analysis for the PMUT finite element model (FEM) was performed using a two-dimensional axisymmetric geometry implemented in COMSOL Multiphysics to determine the mesh density required to identify the resonance frequency of a PMUT element with a radius of 220 μm. The model exploited axisymmetry about the central axis of the PMUT diaphragm. The coupled physics were implemented using the Solid Mechanics and Electrostatics interfaces, which were linked through the Piezoelectric Effect Multiphysics coupling.

Figure S1 shows the maximum out-of-plane displacement observed for a PMUT model with a Ni support structure width of 150 μm. The resonance frequency, identified as the frequency corresponding to the maximum displacement magnitude, was observed to converge to approximately 1.85 MHz as the mesh was refined. Based on this convergence behavior, the model discretized with 11,328 finite elements was selected and used to generate the results presented in **Figure 1** of the main manuscript.

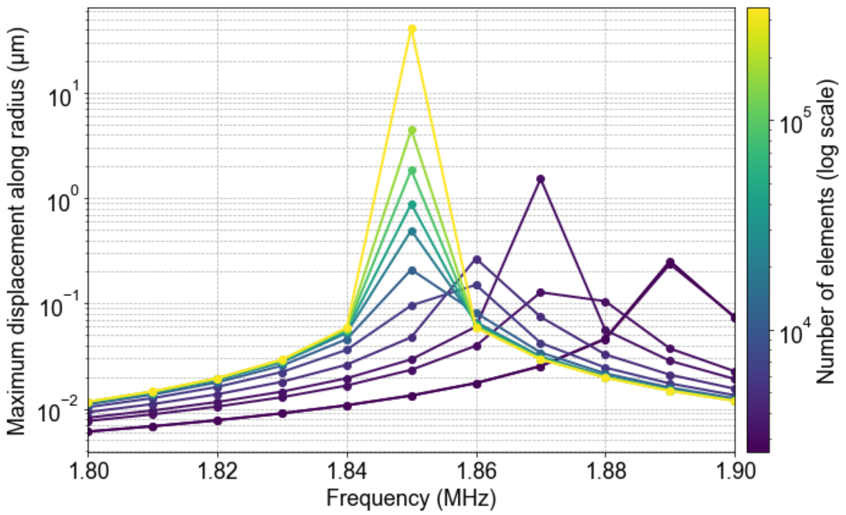

Figure S1. Maximum displacement plotted as a function of frequency sweep for models with different number of elements, for 220 μm radius PMUT element with 100 % top electrode coverage element and 150 μm Ni support structure width.

Table S1 summarizes the basic mechanical properties assigned to the materials explicitly defined in the model. All remaining mechanical properties, as well as the electrical properties of the piezoelectric layer, were taken from the built-in COMSOL material library. The backside Ni plating seed layer, $LaNiO_3$ bottom electrode, and Pt top electrode were neglected in the model to reduce computational complexity.

Table S1. Basic mechanical properties of the materials in the PMUT components

PMUT Components	Materials	Mechanical properties of materials		
		**Elastic modulus (GPa)**	**Density (kg/m³)**	**Poisson's ratio**
Rigid structure for deflection area confinement	Electroplated Ni	219	8900	0.31
Stainless-steel substrate	UNS S30400	209	8000	0.29
Lead Zirconate Titanate (PZT) layer	Lead Zirconate Titanate (PZT-8)	Built-in matrix in COMSOL, e.g., C_{11} = 146.876GPa	7600	N/A*
Contact pad	Gold (Pt)	70	19300	0.44

* Derived from the built-in elastic compliance matrix components in the COMSOL library; typically ranging from 0.3 to 0.3l.

The frequency response of the PMUT was evaluated using a Frequency Domain study. Mechanical boundary conditions were applied within the Solid Mechanics interface, where the bottom surface of the Ni support structure was assigned a Fixed Constraint boundary condition.

Electrical boundary conditions were applied within the Electrostatics interface. A Terminal boundary condition with an applied voltage of 1 V was assigned to the interface between the PZT layer and the substrate, while a Ground boundary condition was applied at the interface between the Au top electrode and the PZT layer.

For the convergence analysis, the two-dimensional PMUT profile was discretized using Free Triangular mesh elements. The parameters, including element size settings, are summarized in **Table S2**. The frequency-domain simulations were solved using the MUMPS (Multifrontal Massively Parallel Sparse Direct Solver) solver with a relative tolerance of 0.001.

Table S2. Basic mechanical properties of the materials in the PMUT components

Maximum element size (μm)	Minimum element size (μm)	Maximum element growth rate	Curvature factor	Resolution of narrow regions	Number of elements
80	6.4	1.8	0.8	1	2610
132	20	2	1	0.9	2717
52	2.4	1.5	0.6	1	3108
40	0.8	1.4	0.4	1	3271
14.8	0.05	1.25	0.25	1	4735
8	0.03	1.2	0.25	1	5861
4	0.008	1.1	0.2	1	11328
2	0.12	1.3	0.3	1	20358
1.35	0.12	1.1	0.3	1	42802
0.9	0.12	1.1	0.3	1	87227
0.65	0.12	1.1	0.3	1	165210
0.32	0.12	1.1	0.3	1	692082
0.23	0.12	1.1	0.3	1	1412788
0.15	0.12	1.1	0.3	1	3447614

2. Finite Element Model with SU8 Support Structure and Modification of Strain-Charge Form Relative Permittivity Tensor Matrix

To compare the bending motion of the PMUT structure with two different support structure materials (Ni and SU-8), an FEM model was created in which the support structure was replaced with SU-8 material properties, as shown in Figure 1(b) of the manuscript. Table S2 summarizes the mechanical properties of the materials used in the SU-8 PMUT components. The model discretized with 11,328 finite elements was selected based on the convergence behavior discussed in the previous section.

In the COMSOL Multiphysics simulation, the built-in relative permittivity tensor is $\varepsilon_{11}^{T} = \varepsilon_{11}^{T}$=1290 and ε_{33}^{T}=1000. To better match the simulation with experimental results, ε_{33}^{T} was adjusted to 283. All results shown in Figure 1 (a), (b), (c) was obtained using this modified permittivity tensor.

Table S3. Basic mechanical properties of the materials in the SU8-support-structure PMUT components

**PMUT Components**	**Materials**	**Mechanical properties of materials**		
		**Elastic modulus (GPa)**	**Density (kg/m^3)**	**Poisson's ratio**
Rigid structure for deflection area confinement	SU-8	4.04	1200	0.22
Stainless-steel substrate	UNS S30400	209	8000	0.29
Lead Zirconate Titanate (PZT) layer	Lead Zirconate Titanate (PZT-8)	Built-in matrix in COMSOL, e.g., C_{11} = 146.876 GPa	7600	N/A*
Contact pad	Gold (Pt)	70	19300	0.44

* Derived from the built-in elastic compliance matrix components in the COMSOL library: typically ranging from 0.3 to 0.3l.
